# The Association Between Periodontal Disease and Acute Coronary Syndrome—A Clinical Analysis

**DOI:** 10.3390/jcm14072447

**Published:** 2025-04-03

**Authors:** Marius Rus, Bianca Maria Negruțiu, Cristian Nicolae Sava, Georgeta Pasca, Felicia Liana Andronie-Cioara, Simina Crisan, Mircea-Ioachim Popescu, Claudia Elena Staniș, Claudia Judea Pusta

**Affiliations:** 1 Department of Medical Disciplines, Faculty of Medicine and Pharmacy, University of Oradea, 410073 Oradea, Romania; 2 Cardiology Department, Bihor Clinical Emergency Hospital, 410169 Oradea, Romania; 3 Department of Dental Medicine, Faculty of Medicine and Pharmacy, University of Oradea, 410073 Oradea, Romania; 4 Department of Psycho Neuroscience and Recovery, Faculty of Medicine and Pharmacy, University of Oradea, 410073 Oradea, Romania; 5 Cardiology Department, “Victor Babes” University of Medicine and Pharmacy, 300041 Timisoara, Romania; simina.crisan@umft.ro; 6 Faculty of Medicine and Pharmacy, University of Oradea, 10 Piața 1 Decembrie Street, 410073 Oradea, Romania; 7 Department of Morphological Disciplines, Faculty of Medicine and Pharmacy, University of Oradea, 410073 Oradea, Romania

**Keywords:** periodontal disease, acute coronary syndrome, prevention, coronary artery disease, diabetes, smoking, cardiovascular risk

## Abstract

**Background:** This study investigated the association between periodontal disease and acute coronary syndrome (ACS), while examining periodontitis as an independent predictor of STEMI. **Materials and Methods:** This study included 166 patients with ACS, of whom 103 had a history of periodontal disease. **Results:** The results showed that patients with periodontal disease were more likely to have a history of tobacco use (78.6%), diabetes (53.1%), and poor oral hygiene (72.8%). This study also found a significant association between periodontal disease and the severity of ACS (STEMI—44.7%, three-vessel/LM disease—30.1%), with patients experiencing more frequent and severe cardiovascular complications (HF—51.6%, valvulopathy and mechanical complications—22.6%, arrhythmias—19.4%). The findings support previous studies suggesting a link between periodontal disease and cardiovascular risk. This study highlights the importance of considering periodontal disease as a potential risk factor for cardiovascular disease and the need for improved access to oral health care to reduce the burden of cardiovascular events. **Conclusions:** Periodontal disease was identified as an independent predictor of STEMI, highlighting the need for periodontal health assessments as part of routine management in cardiovascular risk stratification. The precise mechanisms continue to be an area of active investigation.

## 1. Introduction

Cardiovascular disease (CVD) remains one of the leading causes of mortality in Europe, while periodontal disease is the sixth most common human disease, with a high prevalence of severe periodontitis worldwide [1]. Previous studies have demonstrated the association between periodontal disease and the risk of developing CVDs, including acute coronary syndrome (ACS), heart failure (HF), peripheral artery disease (PAD), atherosclerosis, and stroke [2]. Furthermore, a bidirectional link to diabetes has been suggested, contributing to the increased cardiovascular risk, while smoking represents a shared risk factor for ACS and periodontal disease [2].

Untreated periodontal disease can cause tooth loss through irreversible damage to dental supportive tissues (periodontal ligament, cementum and alveolar bone) [2]. Increased concentrations of pathogenic bacteria within the dental plaque result in a strong immune response, possibly connecting periodontitis to atherosclerotic coronary artery disease [2]. This association is exacerbated by high levels of systemic inflammatory markers, such as C-reactive protein (CRP) and interleukin-6 (IL-6) [3,4]. Increased concentrations of bacterial surface molecules, e.g., lipopolysaccharides (LPS), further stimulates the release of matrix metalloproteinases (MMPs), enzymes responsible for extracellular matrix remodeling and bone destruction [2]. Thus, periodontal pathogens can lead to the destruction of the periodontal pocket epithelium. This allows oral bacterial organisms to directly invade the cardiovascular system (pericardium, heart valves, atherosclerotic plaques) as well as several other organs and tissues through bloodstream dissemination [2].

This chain of events produces platelet aggregation on the one hand and an exuberant inflammatory response that may exacerbate atherogenesis on the other hand [4]. Some individuals appear to have ‘underground’ inflammatory characteristics that place them at increased risk for both atherosclerosis and periodontal disease [4]. Serological studies revealed that elevated periodontal bacteria antibody titers are linked to the development of atherosclerosis [5]. Around eight hundred different species of bacteria have been discovered in human dental plaques [2].

Li et al. further highlights that periodontal disease raises the levels of cytokines, factor X, and prothrombin, leading to exaggerated platelet aggregation and thrombotic clot formation [6]. According to most studies, periodontal disease is an independent activator of the acute phase of inflammation, possibly due to the systemic dissemination of periodontal pathogens and/or endotoxins [2]. This effect leads to an increase in the function of vascular smooth muscle, targeting arteries especially [2]. This systemic inflammation can potentially destabilize atherosclerotic plaques in the coronary arteries, leading to ACS.

This paper aims to investigate the association between periodontal disease and acute coronary syndrome by analyzing differences in clinical characteristics between patients with and without a history of periodontal disease. Our research examined its independent predictive value in the development of ST-elevation myocardial infarction (STEMI), considering a multitude of confounding factors. Thus, we provide novel insights into the strength of periodontal disease as an independent risk factor for severe coronary events. Moreover, the specific demographic, clinical, and dental characteristics that may contribute to this connection are presented, emphasizing the complex interplay between periodontal disease and CVDs. Despite growing evidence supporting the association, the exact pathways remain a subject of ongoing research.

## 2. Materials and Methods

The study was a retrospective, observational cohort study conducted over five months (March–July 2024). The study sample was selected from among the patients who were diagnosed with an acute coronary event in the Cardiology Department of the Bihor County Clinical Emergency Hospital. The study protocol was approved by the ethics committee of Bihor County Clinical Emergency Hospital. All data were obtained through anamnestic means, with no testing imposed upon any of the participants.

**Figure 1:**
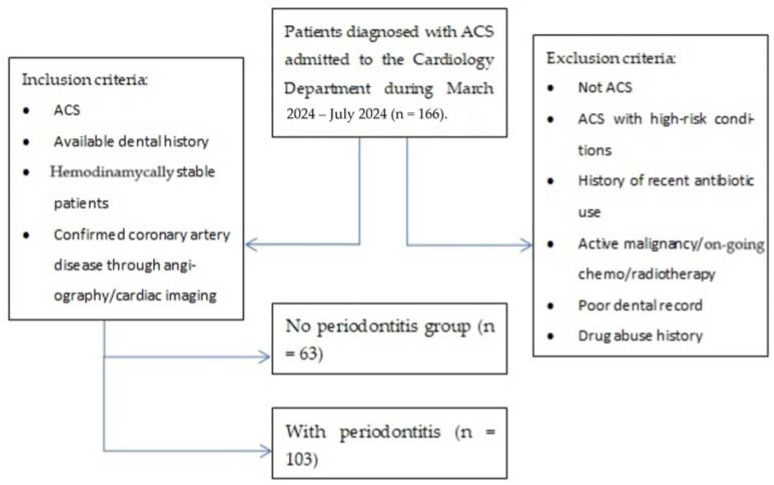
Patient enrollment.

The total sample size was166 patients (86 men, 80 women) aged 31–89 years old. The patients included were diagnosed with an acute coronary event. Figure 1 displays the patient’s enrollment method.

The inclusion criteria were as follows:

- An acute coronary syndrome (ACS) diagnosis (unstable angina, non-ST-elevation myocardial infarction—N-STEMI, ST-elevation myocardial infarction—STEMI) based on the clinical presentation (chest pain over 30 min, dyspnea, diaphoresis, etc.), and paraclinical characteristics (ischemic electrocardiographic findings, with or without elevated cardiac enzyme levels);
- The patients’ electronic charts revealed prior presentations to our hospital’s dentistry department as well as the oral and maxillofacial surgery department. All patients included in the study presented a history of early permanent teeth loss, partial edentulism, gingival bleeding, and dental imaging, with complete periodontal charts;
- Patients were hemodynamically stable patients in order to undergo the necessary assessment and data collection without immediate life-threatening complications;
- Patients who underwent coronary angiography or other cardiac imaging to confirm coronary artery disease.

The exclusion criteria were as follows:

- Patients admitted with suspicions of ACS, which was later denied;
- Patients admitted with ACS with other high-risk conditions (pulmonary embolism, septic shock, cardiac tamponade, etc.) that prevented undergoing coronary intervention during the duration of the study;
- History of recent antibiotic use (last 3 months) given that systemic antibiotics in the past three months could influence periodontal status;
- Active malignancies or patients undergoing chemo/radiotherapy as these conditions affect both CV as well as periodontal health;
- Poor dental records;
- History of drug abuse, particularly cocaine or amphetamines, contributing to ACS through non-atherosclerotic mechanisms.

The participants were divided into two groups:

- Group NP, the group with no periodontitis, consisted of 63 patients (26 men—41.3% and 37 women—58.7%), diagnosed with acute coronary syndrome based on clinical presentations, electrocardiographic findings, and elevated cardiac enzyme levels. Individuals in this group presented no dental history of periodontitis.
- Group WP (with periodontitis) comprised 103 patients (60 men—61.2% and 40 women—38.8%) diagnosed with ACS based on clinical presentations, EKG findings, and cardiac enzyme levels and presenting with a dental history of periodontitis with or without prior treatment.

Variables:

- The presence of periodontal disease was established using visual inspection, periodontal probing, and bone level evaluations on radiography (in the patients’ medical records).
- Signs of periodontal disease included the following: active bleeding of the gingiva in the presence of absence of mild tissue manipulation, gingival retraction, halitosis, radiographic bone loss according to patients’ history, teeth loss, pain, and dental mobility.

The confounding factors considered included age, sex, smoker status, systemic diseases, heart failure, hypertension, coronary artery disease, congenital heart disease, and dyslipidemia.

The presence of heart failure (HF) was established according to the patients’ medical records, as well as the current definition of the pathology (clinical characteristics and paraclinical criteria) in patients with newly diagnosed HF.

High blood pressure (HBP) was defined as persistent elevation of systolic blood pressure ≥ 140 and/or diastolic blood pressure ≥ 90 mmHg.

Congenital heart disease (congenital HD) refers to structural heart abnormalities present since birth, as stated in the patients’ medical records. Most frequently, these included atrial septal defect, bicuspid aortic valve, patent foramen ovale, Wolff–Parkinson–White syndrome, etc.

Systemic disease included the presence of diabetes mellitus, autoimmune disease (such as systemic lupus erythematous, rheumatoid arthritis, psoriasis, etc.), obesity, and other pathologies (thrombophilia, endocrine pathology, Alzheimer’s disease).

The pharmacological treatment mentioned included the acute coronary syndrome medical recommendations according to the current ESC guidelines, along with the patient’s chronic treatment scheme (antidiabetics, antihypertensives, etc.).

All the data from the study were analyzed using IBM SPSS Statistics 25 and illustrated using Microsoft Office Excel/Word 2024. Qualitative variables were written as counts or percentages and were tested between groups using Fisher’s exact test. Z-tests with Bonferroni correction were used to further detail the results obtained in the contingency tables. Univariable and multivariable logistic binomial regression models were used in the prediction of STEMI. Models were tested for significance and goodness of fit. Performance of the prediction was calculated as odds ratios with 95% confidence intervals, along with the significance value. The threshold considered for the significance level for all tests was considered to be α = 0.05.

## 3. Results

### Data Analysis

Data from Table 1 show the characteristics of the analyzed patients according to the existence of periodontitis. Periodontitis was present in 62% of patients, while the remaining 38% presented no dental history of periodontitis.

**Figure 2:**
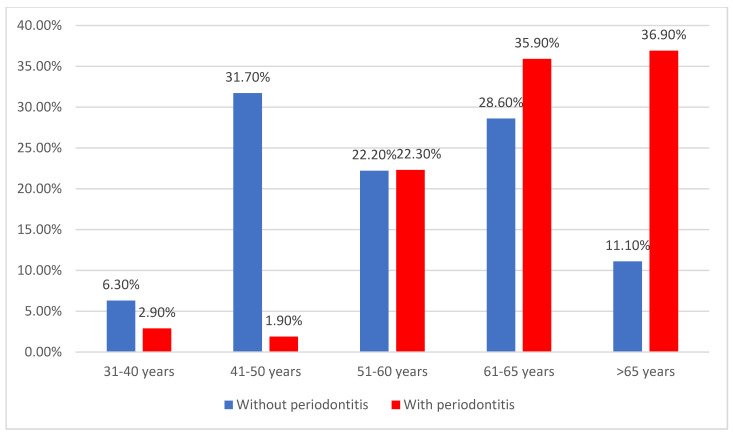
Distribution of patients according to age and the existence of periodontitis.

As displayed in Figure 2, most of the patients were between 6 and 65 years old (33.1%) or above 65 years old (27.1%). Significant differences were observed between groups (*p* < 0.001). Patients with an age between 41 and 50 years presented significantly less frequently periodontitis (31.7% vs. 1.9%), while patients with an age above 65 years presented periodontitis significantly more frequently (36.9% vs. 11.1%).

**Figure 3:**
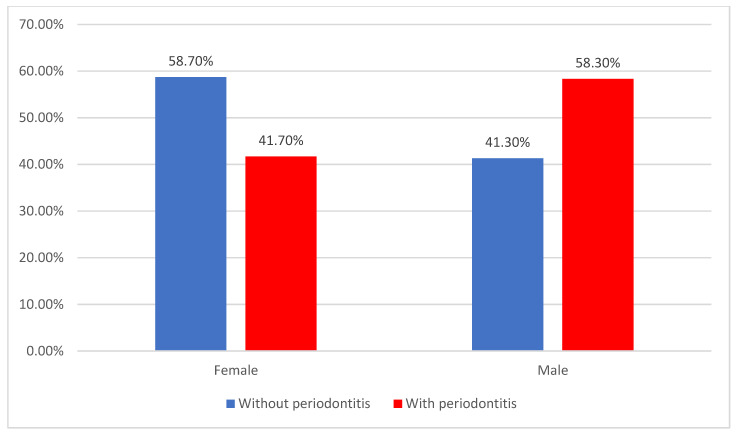
Distribution of patients according to gender and the existence of periodontitis.

Regarding sex, besides being the dominant category diagnosed with ACS in this study (51.8%), men were associated with periodontitis significantly more often (58.3% vs. 41.3%) (*p* = 0.038) (Figure 3).

**Figure 4:**
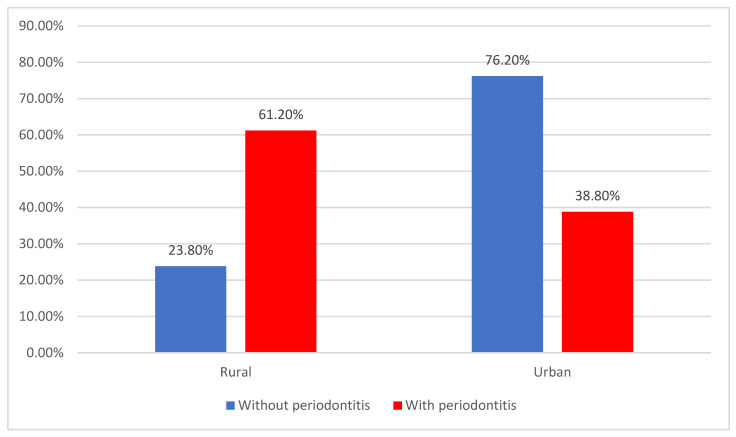
Distribution of patients according to background and the existence of periodontitis.

According to Figure 4, the background distribution shows a majority of patients living in urban environments (53%), with those living in rural environments significantly more associated with periodontitis (61.2% vs. 23.8%) (*p* < 0.001).

**Figure 5:**
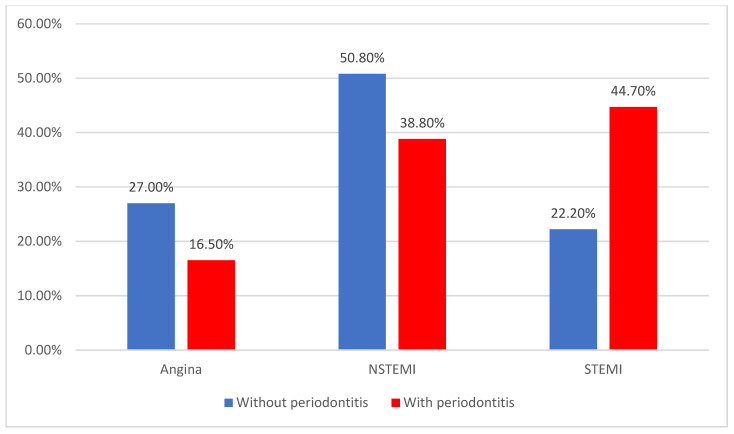
Distribution of patients according to the type of ACS and the existence of periodontitis.

According to the type of acute coronary syndrome, NSTEMI (43.4%) and STEMI (36.1%) were the most frequent among patients, with STEMI being significantly more associated with periodontitis (44.7% vs. 22.2%) (*p* = 0.011) (Figure 5).

Data from Table 2 show the multivariable logistic regression model used in the prediction of STEMI using periodontitis. Results show that, while adjusting for the presence of male gender, smoking, systemic diseases, and the existence of heart failure, periodontitis remains a significant and independent predictor over the existence of STEMI, patients with periodontitis having increased odds of developing STEMI by 2.294 times (95% C.I.: 1.056–4.983) (*p* = 0.036).

In addition, using any other confounding factors (such as age category) for model adjustment will reduce the significance of the model, invalidating the prediction. Considering a list of possible confounding factors besides periodontitis, such as age, gender, background, smoking status, systemic diseases, heart failure, dyslipidemia, congenital heart diseases, valvulopathy, or coronary artery disease, and using the stepwise forward Wald method, only the univariable model with periodontitis remains with a significant prediction (OR = 2.767, 95% C.I.: 1.359–5.633, *p* = 0.005).

**Figure 6:**
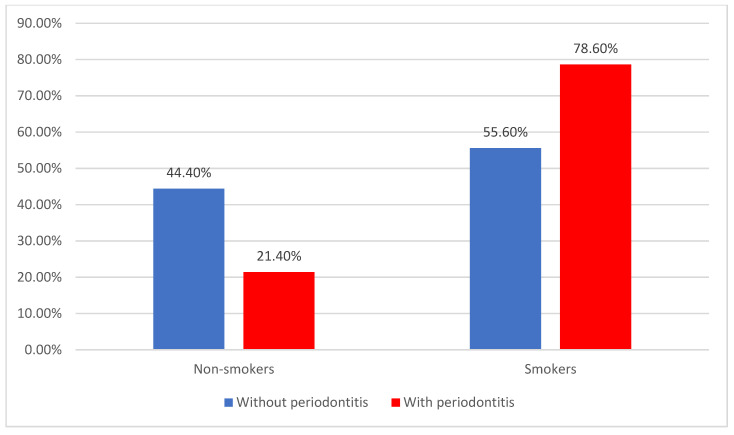
Distribution of patients according to smoking status and existence of periodontitis.

Figure 6 shows the distribution of patients regarding smoking status, with most ACS patients being smokers (69.9%), and ACS is also significantly associated with periodontitis (78.6% vs. 55.6%) (*p* = 0.003).

Systemic diseases were prevalent in most patients (92.2%), and the most frequent ones were diabetes mellitus (51.6%), followed by obesity (22.2%). In total, 8.4% of the patients had congenital heart diseases, 13.9% had valvulopathy, and 16.9% had other cardiovascular conditions, with the most frequent being arrythmias (60.7%), as displayed in in Table 1.

**Figure 7:**
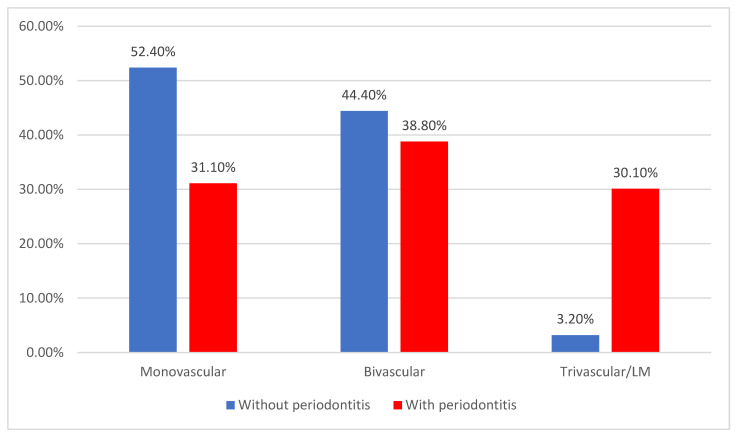
Distribution of the patients according to coronary artery disease and the existence of periodontitis.

Regarding the type of coronary artery disease, monovascular (39.2%) and bivascular (41%) diseases were the most observed (Figure 7). Monovascular coronary artery disease was significantly less associated with periodontitis (52.4% vs. 31.1%), while patients with trivascular/LM localization were significantly more associated with periodontitis (30.1% vs. 3.2%) (*p* < 0.001).

**Figure 8:**
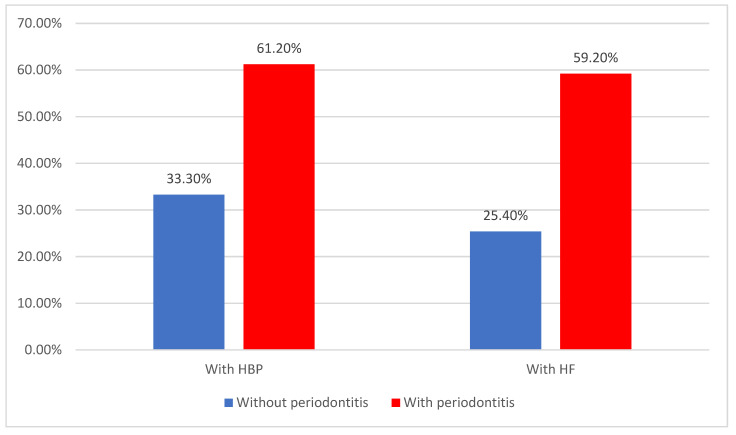
Distribution of the patients according to the existence of high blood pressure, heart failure, and periodontitis.

As displayed in Figure 8, 50.6% of patients already presented high blood pressure before the major coronary event, with 46.4% of patients suffering from heart failure. Both conditions were significantly more associated with an increased risk of ACS, as well as periodontitis (HBP—61.2% vs. 33.3%, *p* = 0.001, HF—59.2% vs. 25.4%, *p* < 0.001).

**Figure 9:**
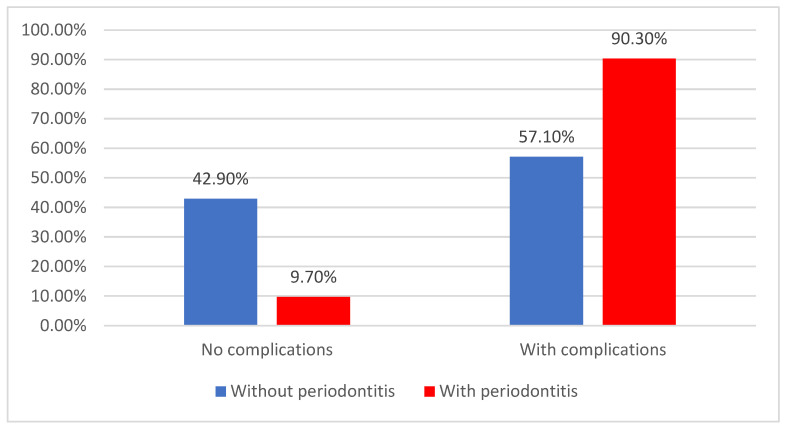
Distribution of patients according to the appearance of cardiovascular complications and the existence of periodontitis.

After the major coronary event, 77.7% of patients developed cardiovascular complications (acute valvulopathy, pulmonary edema, cardiogenic shock, arrythmias, etc.), with the individuals affected by periodontitis being significantly more predisposed to them (90.3% vs. 57.1%) (*p* < 0.001) (Figure 9).

**Figure 10:**
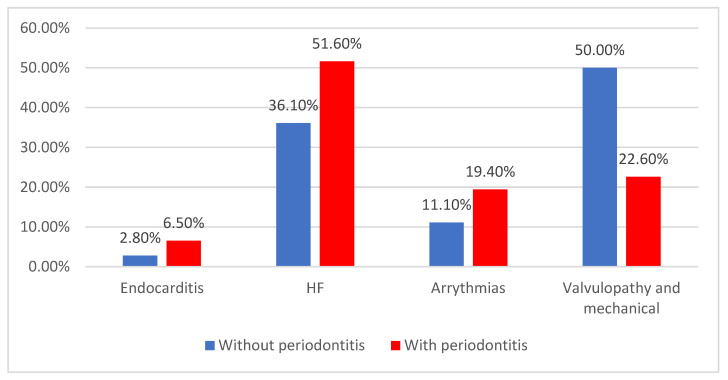
Distribution of patients according to the type of cardiovascular complications and the existence of periodontitis.

Among the type of cardiovascular complications, heart failure (47.3%), valvulopathies, and mechanical complications (30.2%) developed more frequently, with patients with HF being significantly more associated with periodontitis (36.1% vs. 51.6%) (*p* = 0.030) (Figure 10).

Among the dental risks considered in the study population, most patients presented poor dental hygiene (68.1%). The most frequent dental complications of periodontitis encountered in this study were teeth loss (31.9%) and dental mobility (19.9%) or pain (19.9%) (Table 1).

**Figure 11:**
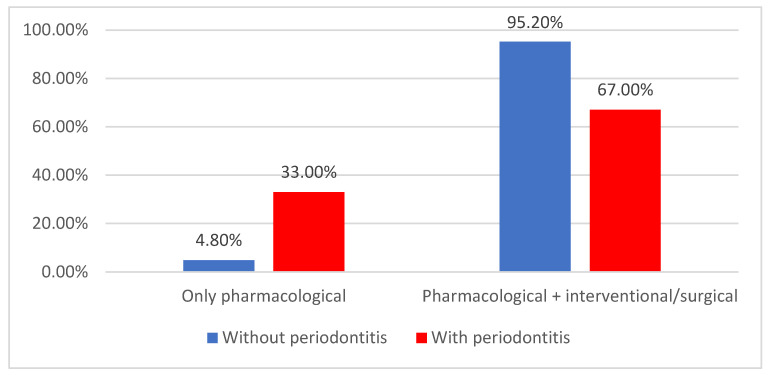
Distribution of the patients according to the type of treatment and the existence of periodontitis.

As exhibited in Figure 11, most patients underwent both pharmacological and interventional/surgical treatments (77.7%). Patients who underwent only pharmacological treatment (due to difficult coronary anatomy, refusal of coronary artery bypass graft surgery, major complications during coronary angiography resulting in the cessation of the intervention, the hemodynamic status of the patient not allowing the procedure to be carried out again, etc.) were significantly more associated with periodontitis (33% vs. 4.8%) (*p* < 0.001).

## 4. Discussion

Our study reports periodontal disease as more prevalent in the older age groups, while men were the most affected by both acute coronary events, as well as periodontitis. These findings are supported by previous studies, suggesting that periodontal disease could be an independent cardiovascular risk factor in men under the age of 60 [5,7,8]. Winning et al. analyzed a sample of 1400 dentate men with periodontal disease over the course of two years. The mean age was 63.7 years old, and coronary heart disease surfaced as more prevalent in the cohort, even though no association regarding CHD incidence could be found [8]. However, another study seems to suggest that focusing solely on prevalent cases of periodontal disease could lead to an underestimation of the incidence of periodontitis and CHD [9].

Disparities in access to oral health care between rural and urban areas have been a topic of discussion for decades. Thus, the results of our study are consistent with numerous epidemiological and public health research displaying a tendency of individuals with poor oral health to reside in rural, low-income, uninsured, or minority populations [10,11,12,13,14]. This social inequality has been increasingly recognized, as the connection to oral disease, such as periodontitis, and general health conditions, especially obesity and diabetes, is becoming more and more clear [11,12]. Common risk factors, including high levels of sugar consumption or cigarettes smoking, become intertwined with untreated chronic infections and active inflammation, even resulting in life-threatening conditions, such as ACS or stroke [15]. Improving access to quality health services, thus integrating oral and primary care health across all backgrounds, could ease the overall burden of cardiovascular events.

According to our study, periodontal disease was associated with the more severe expression of acute coronary syndrome, mainly STEMI, while bivascular coronary artery disease was most frequently encountered on coronarography.

The NHANES III (National Health and Nutrition Examination Trends) study of approximately 5000 patients showed that patients with more severe periodontal disease (with up to two-thirds of gum tissue affected) were up to nine times more at risk of heart attack, as measured by their natural history, than those without periodontal disease. In contrast, patients with milder periodontitis had a 1.5–2.5 times higher risk of heart attack [4]. The same percentage is also reported in the study “The Risk of Dental Atherosclerosis in Communities”, which was carried out on 16,000 subjects. Coronary heart disease was present in people with severe periodontal disease, according to the same author [4]. It is emphasized in this study that the biological basis of the link between periodontal disease and coronary heart disease, although not yet elucidated, is mediated by inflammation, which is related to infectious periodontal disease.

Several studies suggest a strong link between periodontal disease and acute coronary syndrome, especially myocardial infarction [16,17,18,19,20]. Willershausen et al. showed an undeniable association between chronic dental infection and myocardial infarction, while another work by Jansson et al. reported oral health as a possible risk indicator of death attributed to cardiovascular disease when combined with traditional risk factors, such as smoking or diabetes [21,22]. Independent of classic risk factors, the immunological reaction against Porphyromonas gingivalis has been previously associated with myocardial infarction in patients who underwent coronarography [23].

In the CORODONT study, Spahr et al. sought to determine if the prevalence of periodontal pathogens, as well as the pathogen burden in the subgingival biofilm, presented any relevance in the connection between coronary heart disease subjects and periodontitis [24]. A higher concentration of harmful bacteria in dental plaque leads to a notable stimulation of the immune response [2]. Recent studies have detected oral pathogens, such as Aggregatibacter actinomycetemcomitans, Porphyromonas gingivalis, and Prevotella intermedia, in the coronary endothelium, as well as the human atherosclerotic plaque, gaining access to the bloodstream even through intact oral tissue [24]. A. actinomycetemcomitans especially, seems to be acquired at a young age, leading to an early disease debut [24]. Another literature review found that antibody levels of P. gingivalis are associated with atheroma plaque loading and major cardiac events in diabetes patients [23]. The link between periodontitis and atherosclerotic coronary artery disease seems to stem from both the persistent microbial load and the inflammatory response, as evidenced by elevated levels of systemic inflammatory markers like CRP and IL-6 [3,4]. Elevated levels of bacterial surface components, such as LPS, additionally promote the production of MMPs, facilitating extracellular matrix remodeling and bone degradation [2]. Li et al. also observe that individuals with periodontal disease possess increased levels of cytokines, factor X, and prothrombin, resulting in heightened platelet aggregation and the formation of thrombotic clots [6]. Serological research indicates that higher levels of antibodies against periodontal bacteria are associated with the onset of atherosclerosis [5].

However, according to the same work by Voinescu et al., the severity of periodontal disease does not correlate with the severity of heart disease, but the prevalence of severe periodontitis is greater in patients with dilated cardiomyopathy or coronary ischemic disease [23]. Thus, it is neither necessary nor helpful to exaggerate by overestimating the periodontitis–coronary artery disease relationship. In the definition of periodontal disease, the depth of the gingival pocket and the degree of bone loss among other symptoms assess the severity of the disease. Regarding the relationship with atherogenesis and coronary artery disease, bacterial variety, their association, and bacterial load and virulence are also important [4]. Acute coronary syndrome and periodontitis are both multifactorial in nature, with numerous epidemiological studies failing to find a strong connection between the two [25,26].

In our study, smoking was significantly more frequently associated with periodontitis, further supporting the findings of several works. The smoking status is a shared risk factor between myocardial infarction and periodontal disease, according to several studies [2,27]. Beyond the well-established risk associated with cardiovascular disease, smoking seems to also lead to an increase in the inflammatory response to bacterial lipopolysaccharides (LPSs) [27]. Moreover, as it damages neutrophil function, smoking seems to weaken the body’s ability to fight infections associated with periodontal disease, similar to diabetes [27]. Geismar et al. found a strong association between periodontal and cardiovascular disease in patients under 60 years old, with one of the leading risk factors being smoking [28]. These findings are also supported by Dietrich et al., while another study included smoking among the potential effect modifiers of atherosclerosis and periodontal disease link [7,29]. Another study found heavy smoking to be an augmented risk factor for the development of chronic periodontitis [10].

Regarding systemic comorbidities, Roth et al. noted that chronic infections, as well as inflammatory and autoimmune diseases, such as rheumatoid arthritis, psoriasis, systemic lupus erythematosus, and periodontitis, present a significantly higher risk of adverse cardiovascular events [30]. This work supports the hypothesis that a chronic increase in the systemic inflammatory response and burden is causally related to cardiovascular disease development and its consequences [1]. Diabetes mellitus, an important cardiovascular risk factor, was suggested to present a reciprocal association [31]. Poor metabolic control of glucose levels and insulin requirements may follow from chronic infection in periodontal disease. On the other hand, diabetes can lead to several complications, including periodontitis as well as cardiovascular events, with more than a threefold risk increase [31]. Another noteworthy association of periodontitis is the one with Alzheimer’s disease, with inflammation being once again the link between the two entities [31]. Nonetheless, a proper treatment and management plan of periodontal disease positively impacts overall health.

Several studies have shown that poor oral hygiene and dental calculus are related to an increased risk of hypertension [32], and the association displayed in our results between periodontitis and high blood pressure is in line with numerous works over the years. The 2009 to 2018 NHANES study found a significant correlation between periodontal disease and arterial hypertension [33,34]. Interestingly, this association was more relevant among non-smokers and individuals under 45 years of age [33,34]. Another large, French cohort study showed that severe periodontitis was an independent predictor of high blood pressure development [35]. Kawabata et al. studied the risk of hypertension/prehypertension development in young patients suffering from periodontal disease, with an important short-term association being found [36]. At the same time, not all works on the association between oral health and cardiovascular disease found a significant relation between high blood pressure and self-reported periodontal disease [37,38]. The differences in the studies populations, as well as several variables (self-reported disease, clinical measurement, other CV risk factors), need to be considered when identifying the risk of high blood pressure in periodontitis patients.

The development of heart failure represents the consequence of numerous cardiovascular diseases, as well as a leading cause of worldwide morbidity and mortality [2]. As a post-ACS complication in patients with periodontitis in our study, HF was the leading pathology. Several studies have supported the thesis that local and systemic inflammatory factors may correlate periodontal disease to heart failure [39]. Schulze-Späte et al. showed that HF and severe periodontitis were linked more frequently, displaying elevated bone turnover markers compared to control patients [39]. Although the severity of periodontal disease was not associated with HF etiology or symptom severity across all studies, a higher prevalence of periodontal disease in HF patients could not be overlooked by Frohlich et al. [40]. Moreover, another work documented a connection between the two diseases, with individuals with periodontitis displaying higher incidence of HF development [41].

When considering post-ACS complications, valvulopathies and arrythmias were some of the most frequent occurrences observed in our periodontitis patients, followed by endocarditis. The elevated inflammatory response, leading to destabilization of atherosclerotic plaques and resulting in major coronary events, may also create a pro-arrhythmic environment. A large Asian epidemiological study reported a significantly increased incidence of arrythmias, atrial fibrillation (AF) specifically, in subjects with periodontal disease [42]. Furthermore, Hassan et al. emphasized the potential that periodontitis, among other dental infections, may lead to cardiac arrhythmias [43]. A study within the ARIC (Atherosclerotic Risk in Communities) cohort also showed that severe periodontal disease presented an enhanced risk of AF [44], while elevated serum antibody titers against Porphyromonas gingivalis have been linked to late recurrence of AF even in individuals post-ablation [45]. Not only could bacterial pathogens influence the appearance and severity of arrythmias in patients with periodontal disease, the presence of autoantibodies against beta-1 adrenergic receptors in periodontitis individuals may affect the myocardial contractility and heart rate variability, potentially exacerbating arrythmias [46].

In patients presenting both periodontitis and ACS, the systemic chronic inflammation and bacterial burden from periodontal infection may further increase the risk of infective endocarditis (IE). The presence of different pathogens, such as Porphyromonas gingivalis, in the bloodstream may particularly affect patients with valvular heart disease (VHD) [47]. Several findings showed that VHD patients with severe periodontal disease present a greater incidence of IE, with more advanced alveolar bone loss and higher levels of specific periodontal pathogens [48].

According to several studies, periodontal disease patients are generally less likely to follow a treatment scheme consistently [49]. In our study, even though all patients underwent coronarography, periodontitis patients who needed further extensive treatment (i.e., CABG, CTO stenting, multiple angiography procedures) were more predisposed to forego these therapeutical options and chose instead to follow a pharmacological treatment scheme only. Similarly, Amerio et al. observed that high-risk periodontitis patients showed the lowest treatment adherence rates [50]. These lower compliance rates, combined with ongoing smoking habits, could lead to additional cardiovascular complications in ACS individuals, such as acute stent thrombosis, intra-stent restenosis, and other major coronary events. Of course, various demographic factors (age, severity of periodontitis, periodontal surgery, poor oral self-care) further contribute to this hypothesis [49].

Despite the insights provided by our study, several limitations must be acknowledged.

1. Our study establishes an association between periodontal disease and acute coronary syndrome but does not prove causality. Longitudinal or interventional studies are required to determine whether periodontal treatment can directly reduce cardiovascular risk.
2. The study population may not be fully representative of the general population, as it includes only patients who underwent coronary evaluation. This could overestimate the association between periodontitis and cardiovascular disease.
3. The severity of periodontitis was not uniformly assessed using a single standardized diagnostic method across all participants. Variability in the classification of periodontal disease could affect the reliability of the findings.
4. While we accounted for traditional cardiovascular risk factors (such as smoking and diabetes), other unmeasured variables, including genetic predisposition, dietary habits, and socioeconomic status, may influence both periodontal and cardiovascular health.
5. Some risk factors, such as smoking habits and adherence to treatment, were based on self-reported data, which may be subject to recall bias or underreporting.
6. Differences in study populations, sample sizes, and methodologies across various referenced studies may contribute to inconsistencies in the reported strength of the association between periodontitis and cardiovascular disease.
7. The study does not include long-term follow-up to assess how periodontal disease progression influences cardiovascular outcomes over time.

Given these limitations, further research is needed to clarify the causal mechanisms linking periodontal disease to acute coronary events and to evaluate the extent to which periodontal treatment can reduce cardiovascular risk.

## 5. Conclusions

Our study highlights a significant association between periodontal disease and acute coronary events, particularly in men under 60. Periodontitis was linked to more severe acute coronary syndrome and a higher prevalence of bivascular coronary artery disease. Systemic inflammation appears to be a key factor connecting periodontal disease to cardiovascular complications such as myocardial infarction, heart failure, and arrhythmias. Common risk factors like smoking and diabetes further strengthen this connection. Additionally, lower adherence to cardiovascular treatments among periodontitis patients raises concerns for long-term outcomes. Integrating oral healthcare into cardiovascular disease management could help mitigate risks and improve overall health.

## Figures and Tables

**Table 1:** Characteristics of the analyzed patients according to the existence of periodontitis.

Parameter (Nr., %)	Total	No Periodontitis	With Periodontitis	*p*
***N***	166	63 (38%)	103 (62%)	**-**
***Age***				
**31–40 years**	7 (4.2%)	4 (6.3%)	3 (2.9%)	<0.001 *
**41–50 years**	22 (13.3%)	20 (31.7%)	2 (1.9%)	
**51–60 years**	37 (22.3%)	14 (22.2%)	23 (22.3%)	
**61–65 years**	55 (33.1%)	18 (28.6%)	37 (35.9%)	
**>65 years**	45 (27.1%)	**7 (11.1%)**	**38 (36.9%)**	
***Gender (Male)***	86 (51.8%)	26 (41.3%)	60 (58.3%)	0.038 *
***Background***				
**Rural**	78 (47%)	15 (23.8%)	63 (61.2%)	<0.001 *
**Urban**	88 (53%)	48 (76.2%)	40 (38.8%)	
***ACS***				
**Angina**	34 (20.5%)	17 (27%)	17 (16.5%)	0.011 *
**N-STEMI**	72 (43.4%)	32 (50.8%)	40 (38.8%)	
**STEMI**	60 (36.1%)	**14 (22.2%)**	**46 (44.7%)**	
***Risk factors***				
***Smokers***	116 (69.9%)	35 (55.6%)	81 (78.6%)	0.003 *
***Systemic diseases***	153 (92.2%)	55 (87.3%)	98 (95.1%)	0.080 *
***Systemic disease—type***				
**DM**	79 (51.6%)	27 (49.1%)	52 (53.1%)	0.468 *
**Autoimmune**	32 (20.9%)	13 (23.6%)	19 (19.4%)	
**Obesity**	34 (22.2%)	14 (25.5%)	20 (20.4%)	
**Other**	8 (5.2%)	1 (1.8%)	7 (7.1%)	
***Coronary artery disease***				
**Single-vessel disease**	65 (39.2%)	**33 (52.4%)**	**32 (31.1%)**	<0.001 *
**Two-vessel disease**	68 (41%)	28 (44.4%)	40 (38.8%)	
**Three-vessel/LM disease**	33 (19.9%)	**2 (3.2%)**	**31 (30.1%)**	
***HBP***	84 (50.6%)	21 (33.3%)	63 (61.2%)	0.001 *
***HF***	77 (46.4%)	16 (25.4%)	61 (59.2%)	<0.001 *
***Congenital HD***	14 (8.4%)	5 (7.9%)	9 (8.7%)	1.000 *
***Valvulopathy***	23 (13.9%)	6 (9.5%)	17 (16.5%)	0.254 *
***Other CVD***	28 (16.9%)	12 (19%)	16 (15.5%)	0.670 *
***Other CVD—type***				
**Endocarditis**	9 (32.1%)	3 (25%)	6 (37.5%)	0.296 *
**Pericarditis**	2 (7.1%)	2 (16.7%)	0 (0%)	
**Arrythmias**	17 (60.7%)	7 (58.3%)	10 (62.5%)	
***Dyslipidemia***	141 (84.9%)	55 (87.3%)	86 (83.5%)	0.656 *
***Dental risk factors***				
**Poor hygiene**	113 (68.1%)	38 (60.3%)	75 (72.8%)	0.122 *
**Misalignment**	53 (31.9%)	25 (39.7%)	28 (27.2%)	
***CVD complications***	129 (77.7%)	36 (57.1%)	93 (90.3%)	<0.001 *
***CVD complications—type***				
**Endocarditis**	7 (5.4%)	1 (2.8%)	6 (6.5%)	0.030 *
**HF**	61 (47.3%)	13 (36.1%)	48 (51.6%)	
**Arrythmias**	22 (17.1%)	4 (11.1%)	18 (19.4%)	
**Valvulopathy and mechanical**	39 (30.2%)	**18 (50%)**	**21 (22.6%)**	
***Dental complications***				
**Dental mobility**	33 (19.9%)	11 (17.5%)	22 (21.4%)	0.505 *
**Gingival bleeding**	26 (15.7%)	13 (20.6%)	13 (12.6%)	
**Gingival retraction**	21 (12.7%)	10 (15.9%)	11 (10.7%)	
**Pain**	33 (19.9%)	11 (17.5%)	22 (21.4%)	
**Teeth loss**	53 (31.9%)	18 (28.6%)	35 (34%)	
***Treatment***				
**Pharmacological**	37 (22.3%)	3 (4.8%)	34 (33%)	<0.001 *
**Pharmacological + interventional/surgical**	129 (77.7%)	60 (95.2%)	69 (67%)	

* Fisher’s exact test.

**Table 2:** Multivariable logistic regression model used in the prediction of STEMI using periodontitis.

Parameter/Model *	OR (95% C.I.)	*p*
**Periodontitis**	2.294 (1.056–4.983)	**0.036**
**Gender (Male)**	1.473 (0.750–2.893)	0.261
**Smoking**	1.376 (0.641–2.956)	0.413
**Systemic diseases**	0.884 (0.245–3.184)	0.850
**Heart failure**	1.347 (0.675–2.684)	0.398

* Omnibus test of model coefficient: χ^2^ (5) = 11.469, *p* = 0.043, Nagelkerke R^2^ = 0.091, Hosmer and Lemeshow test, *p* = 0.349.

## Data Availability

All data are available in the archive (data base) of the Clinical County Emergency Hospital of Oradea, Oradea, Bihor County, Romania.
